# Atypical Presentation of Necrotizing Sialometaplasia of the Hard Palate: A Diagnostic Dilemma

**DOI:** 10.7759/cureus.42825

**Published:** 2023-08-01

**Authors:** Mohd Faizal Abdullah, Muhd Amir Ridzuan Hamzah, Fattirah Auni Fauzi, Anani Aila Mat Zin, Bazli M Yusoff

**Affiliations:** 1 Oral and Maxillofacial Surgery, Hospital Universiti Sains Malaysia, Kota Bharu, MYS; 2 Oral and Maxillofacial Surgery, School of Dental Sciences, Universiti Sains Malaysia, Health Campus, Kota Bharu, MYS; 3 Pathology, School of Medical Sciences, Universiti Sains Malaysia, Kota Bharu, MYS; 4 Radiology, Hospital Universiti Sains Malaysia, Kota Bharu, MYS

**Keywords:** differential diagnosis, self-limiting lesion, palate, ischemic changes, necrotizing sialometaplasia

## Abstract

Necrotizing sialometaplasia refers to a benign, uncommon, and self-limiting inflammatory reaction concerning the salivary gland tissue, which both clinically and histologically may be easily mistaken for mucoepidermoid carcinoma or squamous cell carcinoma. This may cause irrelevant surgical intervention. Minor salivary glands are the most commonly affected salivary gland, with the hard palate being the most usual site. However, it can involve the other areas in which salivary gland tissue is present in the other oral subsites and pharyngeal areas. Due to the lack of knowledge about this entity and its histological similarities with carcinomas, particularly mucoepidermoid carcinoma, the differential diagnosis of this lesion is difficult. Local ischemia is thought to be the primary cause, leading to the pathogenesis of necrotizing sialometaplasia, and the infiltration of local anesthesia following dental procedures at the palatal region is the leading cause.

## Introduction

Necrotizing sialometaplasia refers to an uncommon inflammatory, benign lesion affecting the salivary glands, and it has a predilection for the palatal region, in which two-thirds of cases are confined to the unilateral lesions. It was first defined by Abrams et al. in 1973 [1]. Although the pathogenesis of necrotizing sialometaplasia is still not definite, it is widely known that it can be caused by a local vascular obstruction that will progress to ischemic necrosis of salivary glands [2].

Due to the lack of understanding of this lesion and its histopathological similarities with carcinomas, with mucoepidermoid carcinoma in particular, the diagnosis of this lesion is challenging [3]. Necrotizing sialometaplasia is only reported in 0.03% of biopsied oral lesions, with a predominance in Whites and an age prevalence between 17 and 80 years, with slight male predominance at a 2:1 ratio [4,5]. The lesion usually starts with localized swelling that subsequently disintegrates into an ulcerative lesion with a non-indurated base [6]. Moreover, the possibility of mistaken interpretation is the most concerning, and squamous cell carcinoma and mucoepidermoid carcinoma are the frequent histological mimics [7].

## Case presentation

**Figure 1:**
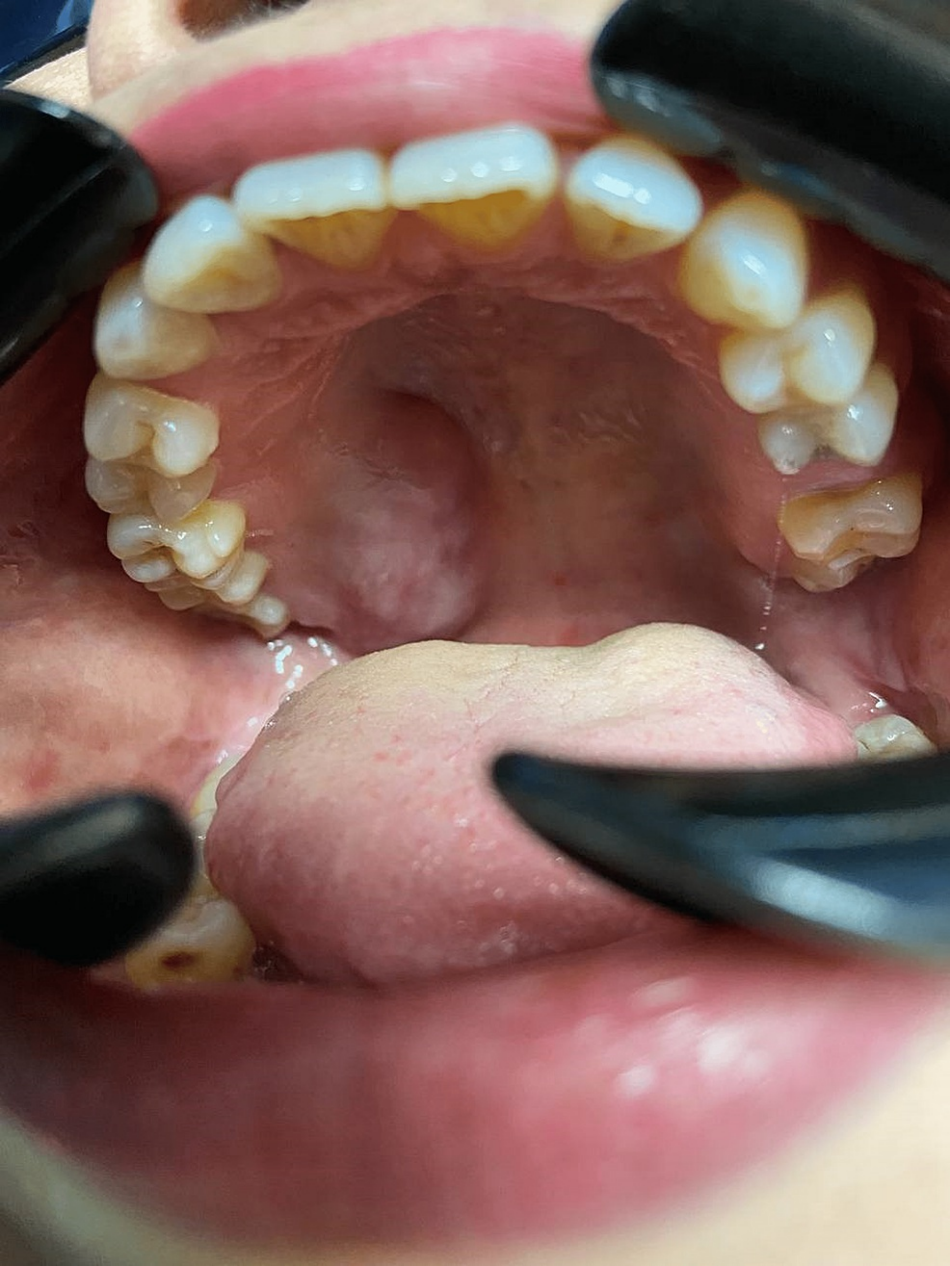
Soft to firm consistency of swelling at the hard and soft palate junction with paresthesia and pain.

A 50-year-old female was recommended by a general practitioner due to a three-month history of swelling at the junction of the hard and soft palate on the right side. The swelling was associated with pain and paresthesia of the hard palate region, and the pain was referred to her right ear. The patient did not have any underlying medical illness or allergies. She denied any trauma or dental extraction prior to the swelling episode. Extraoral examination revealed no neck nodes palpable and no facial asymmetry or swelling. Intraoral examination revealed a 3 × 4 cm swelling at the right soft and hard palate junction, soft to a firm consistency, slight tenderness on palpation, and numbness around the right hard palate region. All teeth are non-carious and vital on cold test and electric pulp test with no periodontal diseases as well (Figure 1).

**Figure 2:**
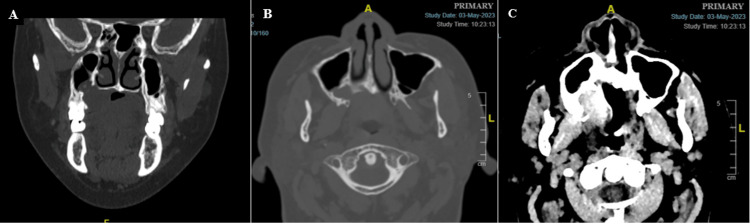
A, B: The coronal and axial section displays bony erosion at the right floor of the maxillary sinus and the hard palate’s right side with an extension of the lesion into the maxillary sinus. C: Presence of ill-defined, peripherally enhancing, hypodense soft tissue lesion at the margin of the soft and hard palate, more on the right side.

The differential diagnosis includes minor salivary gland tumors, such as squamous cell carcinoma, mucoepidermoid carcinoma, Warthin’s tumor, and adenoid cystic carcinoma [3]. A contrast-enhanced computed tomography (CECT) scan was done, revealing the presence of an ill-defined peripherally enhancing hypodense soft tissue lesion at the margin of the soft and hard palate, more on the right side. Bony erosion was observed at the maxillary sinus floor and the right lateral aspect of the hard palate. Furthermore, the lesion also extends minimally into the maxillary sinus (Figure 2).

**Figure 3:**
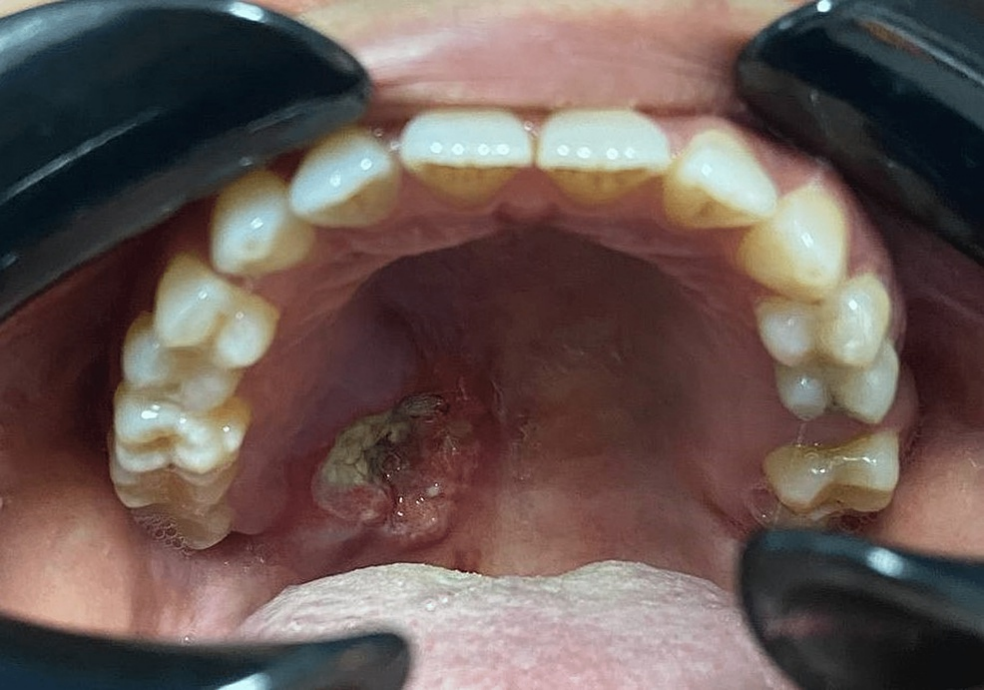
Malignant-looking ulceration one week post incisional biopsy.

The clinical and radiographic impression suggested that the soft tissue tumor of the palate caused bony erosion. An incisional biopsy under local anesthesia was done after the CECT scan to confirm the diagnosis. During the incision, a yellowish fluid came out from the lesion and was sent for culture and sensitivity. A 1 × 1 cm soft tissue was taken at the junction of the soft and hard palate region, and the wound was closed using resorbable suture Vicryl 3/0. Post-biopsy review, the patient criticized an ulcerated lesion over the palate area. Upon examination, there was a crateriform ulcerated lesion with an indurated, rolled-out border, necrotic center, and erythematous margin. There was suspicion of malignancy based on the appearance of the ulcer (Figure 3).

**Figure 4:**
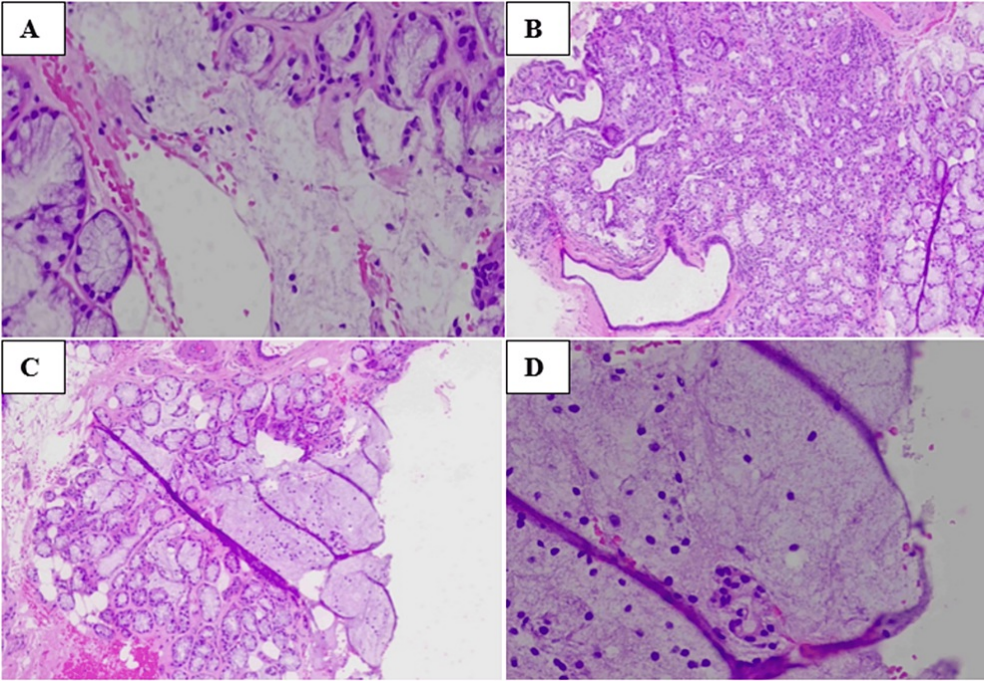
HPE: A: The tissue partly lined by stratified squamous epithelium exhibited pseudoepitheliomatous hyperplastic changes. B: The section illustrates lobules of salivary glands ducts and acini. C: Few lobular and acinar cell necrosis was seen. D: Areas of acini necrosis comprise small pools of mucin enclosed by mixed inflammatory cells, predominantly lymphocytes. HPE: histopathological examination

Histopathological examination (HPE) (Figure 4) displayed fibrocollagenous tissue composed of lobules of the salivary gland’s duct and acini. The tissue partly lined by stratified squamous exhibits pseudoepitheliomatous hyperplasia changes, and the underlying connective tissue displayed few lobular and acinar cell necrosis associated with squamous metaplasia with the architecture preservation. Areas of acini necrosis comprise a small pool of mucin enclosed by mixed inflammatory cells, predominantly lymphocytes, with no microorganism, definitive cellular atypia, or malignancy observed. An additional test was also performed, including cluster of differentiation 117 (CD117), cytokeratin 7, Ki-67, periodic acid-Schiff (PAS), p63, smooth muscle actin (SMA), PAS-diastase, and Southgate’s mucicarmine.

**Figure 5:**
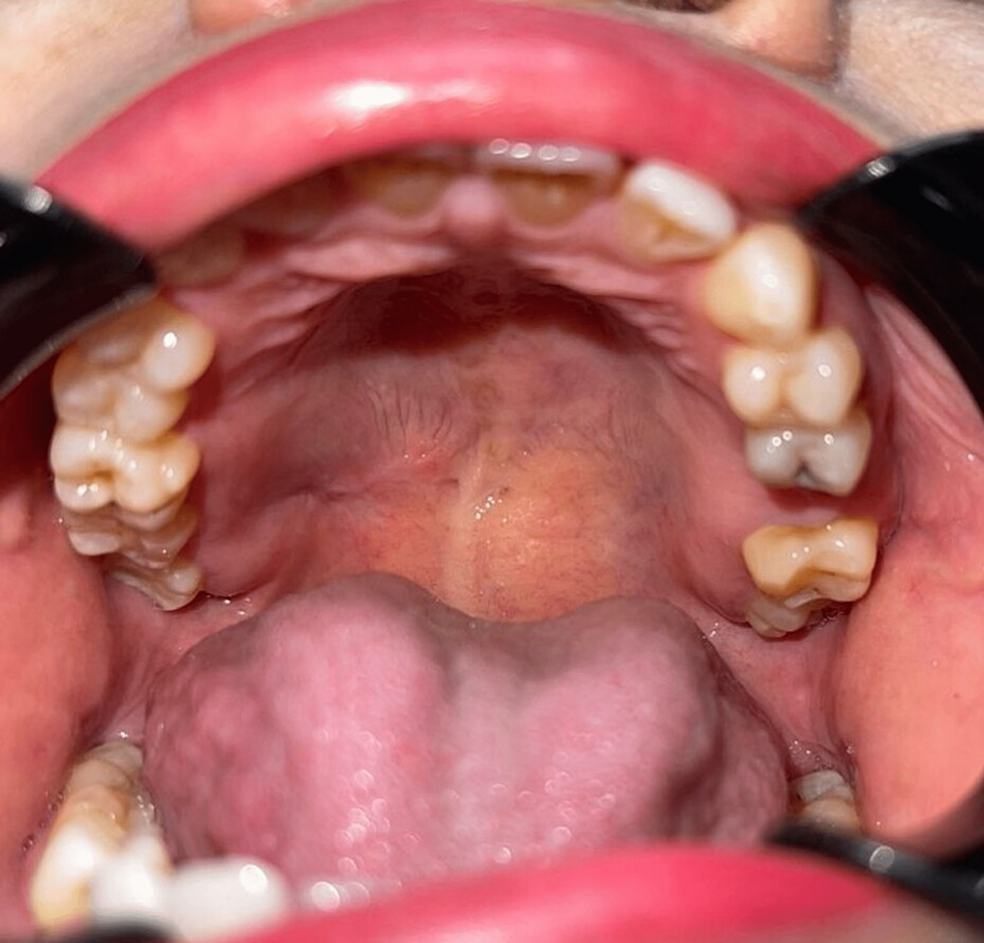
Well-healed post-incisional biopsy site.

Blood investigations showed no significant findings. Pus culture and sensitivity test revealed mixed organism growth. After a period of three weeks, the patient presented with a healing area of ulceration and no paresthesia and pain in the hard and soft palate region (Figure 5).

## Discussion

Anneroth and Hansen asserted that the etiology of necrotizing sialometaplasia may be categorized into five stages: infarction, sequestration, ulceration, reparation, and healing [8]. The severity and extent of the injury depend on the host tissues’ ability to repair, and these several stages may occur simultaneously in various locations. In our case, although the patient denied any local trauma to the palatal region, we assumed that the patient might have had a microtrauma to the palatal region that led to the infraction and slow sequestration of the necrotic material. This leads to the appearance of soft to firm swelling and the presence of local infection. Considering the chronicity of the swelling and the presence of local infection, there were compression of the greater palatine nerve and bony erosion of the maxillary sinus floor and right lateral aspect of the hard palate.

There are many risk factors that have been identified to explain how local ischemia of the palate first develops. The most common of them is thought to be local trauma. Injecting local anesthetic with adrenaline into the hard palate prior to maxillary tooth extraction may cause ischemia by pharmacologic vasoconstriction [9]. Radiation, allergies, respiratory conditions, past adenoidectomy procedures, and nearby tumors may cause compression, resulting in ischemia [5]. Additionally, according to Senapati et al., there have been recent indications that necrotizing sialometaplasia could potentially manifest as a sign of localized vasculitis [10]. In addition, a combination of chemical and mechanical variables, for instance, the low pH of the gastric contents and using fingers to mechanically cause vomiting, are possible predisposing factors that have also been mentioned in the literature. These include chemical irritation brought on by bulimia and persistent vomiting [11]. Because of the minimized immunity defense and anemia, which increases blood viscosity that favors the ischemic occurrence, some systemic diseases such as HIV and diabetes mellitus are considered predisposing factors [12].

The relevance of necrotizing sialometaplasia is observed in its histological and clinical similarity to malignancy. In our case, clinical and radiographic assessments initially revealed a possible diagnosis of malignant tumors, given the signs and symptoms of paresthesia and bony erosion of the maxillary sinus and palatal bone [13]. To our knowledge, most of the radiographic investigations, such as magnetic resonance imaging, were performed after the incisional biopsy. Pre-biopsy CECT imaging of necrotizing sialometaplasia was only reported at the stage of ulceration of necrotizing sialometaplasia [11,12], not at the swelling stage; this creates a dilemma for us since mandatory biopsy is compulsory to establish a diagnosis. The post-biopsy malignant-looking ulcerative appearance of necrotizing sialometaplasia in our case also creates panic in the patient and clinician. However, the diagnosis can only be established by the final histopathological report. The presence of bony erosion is extremely rare in necrotizing sialometaplasia cases, and bone erosion was only reported confined to the hard palate area [14]. In our case, both the palatal bone and maxillary sinus floor were affected, making it an atypical presentation of necrotizing sialometaplasia. A thorough clinical history and a well-focused biopsy section are required for the difficult necrotizing sialometaplasia diagnosis. The most representative sample is obtained from a biopsy of the ulcer’s base and its most indurated and elevated edge [15]. However, in our case, defining the most suitable site for incisional biopsy is difficult. The confirmatory diagnosis is frequently made using a combination of histological and clinical evidence. Immunohistochemistry can further supplement the diagnosis, as demonstrated in our case, but the gold standard for histopathologically identifying necrotizing sialometaplasia remains hematoxylin and eosin staining [16].

## Conclusions

Since necrotizing sialometaplasia frequently progresses rapidly in the initial few weeks to months, it may be misinterpreted as a cancerous lesion. Numerous patients have had unnecessary interventions, ranging from conservative excision to total maxillectomy. Before more extensive treatment regimens are implemented, it is crucial to recognize necrotizing sialometaplasia. This is due to the fact that it is a benign, self-limiting condition that acquires conservative treatment.
